# Validation of the person-centred coordinated care experience questionnaire (P3CEQ)

**DOI:** 10.1093/intqhc/mzy212

**Published:** 2018-12-01

**Authors:** Helen Lloyd, Ben Fosh, Ben Whalley, Richard Byng, James Close

**Affiliations:** 1 School of Psychology/Cognition institute, University of Plymouth, UK; 2 University of Plymouth Faculty of Medicine and Dentistry, John Bull Building, Plymouth Science Park, Research Way, Plymouth, UK; 3 Plymouth University, Peninsula Schools of Medicine and Dentistry, NIHR CLAHRC, South West Peninsula (PenCLAHRC), 1 Davy Road, Plymouth Science Park, Plymouth, UK

**Keywords:** quality measurement, quality management, quality improvement, quality management

## Abstract

**Background:**

Measuring patient experiences of healthcare is increasingly emphasized as a mechanism to measure, benchmark and drive quality improvement, clinical effectiveness and patient safety at both national and local NHS level. Person-centred coordinated care (P3C) is the conjunction of two constructs; person-centred care and care coordination. It is a complex intervention requiring support for changes to organizational structure and the behaviour of professionals and patients. P3C can be defined as: ‘care and support that is guided by and organized effectively around the needs and preferences of individuals’. Despite the vast array of PRMS available, remarkably few tools have been designed that efficiently probe the core domains of P3C. This paper presents the psychometric properties of a newly developed PREM to evaluate P3C from a patient perspective.

**Methods:**

A customized EMIS search was conducted at 72 GP practices across the South West (Somerset, Devon and Cornwall) to identify 100 patients with 1 or more LTCs, and are frequent users of primary healthcare services. Partial Credit Rasch Modelling was conducted to identify dimensionality and internal consistency. Ecological validity and sensitivity to change were assessed as part of intervention designed to improve P3C in adults with multiple long-term conditions; comparisons were drawn between the P3CEQ and qualitative data.

**Results:**

Response rate for the P3CEQ was 32.82%. A two-factor model was identified. Rasch analysis confirmed unidimensionality of each factor (using infit MSQ values between 0.5 and 1.5). High internal consistency was established for both factors; For the Person-centred scale Cronbach’s Alpha = 0.829, Person separation = 0.756 and for the coordination scale Cronbach’s alpha = 0.783, person separation = 0.672.

**Conclusions:**

The P3CEQ is a valid and reliable measure of P3C. The P3C is considered to have strong face, construct and ecological validity, with demonstrable sensitivity to change in a primary healthcare intervention.

## INTRODUCTION

Measuring patient experiences of healthcare is increasingly emphasized as a mechanism to measure, benchmark and drive quality improvement, clinical effectiveness and patient safety at both national and local NHS levels [1–4]. Research has established that patient experiences of care are consistently and positively associated with measures of patient safety and clinical effectiveness across a range of disease groups, study designs, settings, population groups and measures [5]. Furthermore, patient experience is also being measured in order to assess new models of care which aim to be person centred and coordinated, both in the UK (e.g. Vanguard, Pioneer, System Transformation Plans) [6] and overseas (e.g. Sustain/Selfie) [7, 8].

Questionnaires that measure patient experiences (Patient Reported Experience Measures—PREMS) are a sub-category of Patient Reported Measures (PRMs) that probe individual patient perspectives on a range of health and social care related experiences. A recent literature synthesis defined the broad construct as a person’s experience of the events that occur across the continuum of care, focusing on tailoring of services to meet needs and engage people as holistic partners in care [9]. The emphasis on experiences of care over a continuum, combined with holistic (tailored) care and care partnerships, highlights the intrinsic links between the principles of person-centred care and patient experience [10, 11].

### Person-centred coordinated care

Person-centred coordinated care (P3C) is the conjunction of two constructs; person-centred care [12] and care coordination [13]. Broadly, P3C can be defined as ‘Care and support that is guided by and organized effectively around the needs and preferences of individuals’ [14]. P3C is a complex system of care incorporating changes to organizational structure and the behaviour of professionals and patients [14]. Person-centred care is underpinned by a set of defined philosophical and practical principles based on the individual’s right to self-determination [11, 15, 16] and collaborative approaches to care planning with patients [17]. We have identified 5 core domains of P3C (which can be delineated into subdomains) [18, 19]. Domains and subdomains are presented in Table 1.

**Table 1:** Domains of P3C.

Primary domains	Subdomains
Information and communication processes	Consistency of contact
	P3C behaviour and Communication
	Knowledge of patient/familiarity
	Information gathering/sharing
Care planning	Care plans
	Case Manager/Key person
	Care coordination (within and across teams)
	Generic care planning
	Single point of contact
Transitions	Continuity of care
Goals and outcomes	Goal setting
	Empowerment
	Self-management
	Carer Involvement
Decision making	Involvement in decision making

These domains correlate with the independent findings of others, suggesting a convergence of the core issues that P3C seeks to address both from the perspective of patients [20], policy makers [21, 22] and research [11, 23].

For person-centred coordinated care to be achieved, we propose that care interactions are guided by consideration of a person’s life context and capabilities. This is achieved via an identification of their resources (both individual and contextual) with this information incorporated into the coordination of that individual’s care across the services they require (see Table 2). This implicitly posits an active role for the person in collaborative and co-managed care.

**Table 2:** A detailed definition of person-centred coordinated care.

Person-centred care	**The co-creation of care between the patient, their family, informal carers, and health professionals.** This definition is becoming widely used by many international organizations including the World Health Organization (WHO), and has been translated into a proven approach used at the Gothenburg University Centre for Person-Centred Care (GPCC). Person-centred care strives to see an individual as bio-psycho-social whole, as a person and not an illness or a collection of conditions.
Capabilities and Resources (of the individual and their wider context)	**The resources and capabilities of the individual/support network (psycho-social, physical, familial) and the wider environmental resources that are non-clinical and in the community.** The latter is referred to as ‘Community-centred approaches’. These complement other types of interventions that focus more on individual care and behaviour change, or on developing sustainable environments whilst acknowledging the importance of social capital for health and wellbeing to flourish.
Coordinated care	Care coordination that is the deliberate combining, in the necessary forms and sequence, of patient care activities by three or more participants (including the patient) so as to deliver the healthcare with the patient. **From a person or family perspective, care coordination is any co-operative activity that helps ensure that the individual’s needs and preferences for health services (and hence information sharing) across people, work-groups, organization and sites are met over time.**

Despite the vast array of PRMS available, there are remarkably few efficient tools that probe the core domains of P3C as a unified construct [18], and that can be easily used to feedback results to practice based settings. This work was part of a programme to develop a consistent framework for evaluating person-centred coordinated care [14] in the UK. We worked with a range of stakeholders including patients, commissioners and service delivery professionals to co-design a brief measure to evaluate person-centred coordinated care from the perspective of the patient [19]. The Person-Centred Coordinated Care Experience Questionnaire (P3CEQ) is an ten-item measure that probes the core domains of P3C; Table 1: Domains of P3C.

The questionnaire is a further development the LTC-6 [19] to cover the multi-faceted construct of P3C designed to be broken down into two dimensions, person-centred care and care coordination. Use of the LTC-6 and additional items were selected through iterative patient workshops. Selected items were subject to cognitive interviewing to ensure items were acceptable and understood as intended. A detailed outline of item generation and selection protocols can be found at [19].

In this paper, we present the results of the next stage of the development of this measure: the psychometric validation against people with Long-Term Conditions (LTCs) in a UK primary care setting.

## METHODS

### Patients

Ethical clearance was obtained from the Faculty Research Ethics Committees (FREC) and the Health Research Authority (HRA). A customized electronic search was conducted at 72 General Practices across the South West of England to identify 100 patients over the age of 18 with one or more LTCs, who were frequent users of general practice (6+ consultations in previous 12 months). Identified patients were invited to complete the P3CEQ.

### Protocol and data collection

All patients identified by the search received an envelope containing a written information sheet, consent form, the P3CEQ, and a tick-box list of LTCs. Patients were asked to complete the form based on the care they had received within the last six months.

Patients were expected to self-complete the form, ensuring their answers reflected personal experiences of P3C. An introductory paragraph described the purpose of the questionnaire—e.g. participants experience and understanding of the care they received from their health and social care team, with ‘care’ referring to any treatment or support received in relation to their health or wellbeing.

### Questionnaire validation

#### Item validation

Items were assessed based on standard criteria including unidimensionality, internal consistency, item internal consistency (IIC) and item discriminant validity (IDV) [24]. Unidimensionality was assessed through Rasch INFIT statistics [25] generated using the Partial Credit Rasch Model, and principle component analysis (PCA) [26]. The Partial Credit Rasch Model is considered suitable for analysis of measures containing both dichotomous and polytomous/ordinal responses [27]. Partial Credit Rasch analysis was conducted using the eRm package for R [28]. The script used to perform the Partial Credit Rasch analysis, and the output is included within the supplementary documentation. Internal consistency was assessed through Cronbach’s alpha, and person-separation reliability. Person-separation reliability is considered an appropriate alternative to Chronbach’s Alpha when data is not continuous [27]. IIC and IDV concern the relationship of each item to its hypothesized scale or domain. For IIC to be acceptable the item should correlate *r* ~≥0.4 with its adjusted scale score. For IDV, the item should have the highest correlation with its scale, in comparison with other scales in the questionnaire [24].

The relationship between demographics, sociocultural levels, and number of medical conditions, to P3CEQ score was analysed using Analysis of Variance, Mann–whitney *U*, and Pearson’s *R*.

P3CEQ data was collected as part of the evaluation of a cross-team collaborative intervention to improve care coordination. Data was examined to determine the sensitivity of the tool to changes in care over time. The validity of identified changes were confirmed by comparison to qualitative data collected during the same intervention.

Items with response rates below 85% were excluded from the validation analysis to ensure the quality of data.

## RESULTS

The P3CEQ was sent to 7200 participants’ across practices in the South West of England of which 2363 were returned, resulting in a response rate of 32.82%, similar to other comparable instruments [29]. The demographics of the participants appear in Table 3. Item 8 was scored as an average of Q8a–Q8d sub-items. Item 4 was excluded from the validation process due to missing data >15%.

**Table 3:** Respondent demographics for the P3CEQ.

Age group			Gender			Education			Number of self-reported LTCs		
	*N*	%		*N*	%		*N*	%		*N*	%
≤24	9	0.4	Male	1030	43.6	None	28	1.2	1	472	20
25-34	37	1.6	Female	1273	53.9	Primary	55	2.3	2	538	22.8
35-44	60	2.5	Other	2	0.1	Secondary	813	34.4	3	438	18.5
45-54	147	6.2	Pref. not to say	2	0.1	College	654	27.7	4	309	13.1
55-64	346	14.6	Missing	56	2.4	Undergraduate	259	11	5	189	8
65-74	669	28.3				Postgraduate	187	7.9	6	119	5
75–84	753	31.9				Missing	367	15.5	≥7	138	5.8
≥85	321	13.6							0/Missing	160	6.8
Missing	21	0.9									

### Sample characteristics

Of the 2363 participants 1030 (43.6%) were male, 1273 (53.8%) were female and 56 (2.4%) were unspecified. Participants’ age was skewed towards an elderly population (skewness = −1.285, SE = 0.050). The modal age group was 75–84. Educational demographic patterns demonstrate a tendency towards more highly educated individuals with 186 (7.9%) of respondents having postgraduate qualification, compared to 28 (1.2%) respondents having no formal education resulting in a slight negative skew (skewness = −0.607, SE = 0.050). The modal educational attainment was secondary school level. For a full demographic table, see Table 3.

### Internal validity

Item 4 exceeded the acceptable missing response threshold and was therefore removed from the measure. Due to the conceptual importance, it has been included as an optional item, but is not included in the P3CEQ scoring system. The final P3CEQ contains 10 items. A two-factor measure was determined by principle component analysis (see Table 4). Dimensions were named after their item content and reflect the conceptual development outlined by Sugavanam *et al.* [19]. Overall scalability was good with the Partial Credit Rasch analysis indicating good fit for each dimension. For the purpose of evaluation, the items were scored as 0,1,2,3 for ordinal/polytomous items and 0,1 for binary items. When generating item scores however, dichotomous items should be scored as 0,3 to ensure equal weighting between ordinal/polytomous items and dichotomous items. IIC and IDV were satisfactory for the majority of items [24], however due to an intrinsic link between care coordination and person-centred care, some items load on both care coordination and person centredness, resulting in low IDV values for those items (see Tables 5 for further detail).

**Table 4:** Principle component analysis (Varimax rotation) of P3CEQ items

Rotated component matrix		
	Person-centredness	Care-coordination
Q1	0.774	0.108
Q2	0.777	0.089
Q3	0.71	0.068
Q5	0.467	−0.045
Q6	0.705	0.243
Q7	0.058	0.727
Q8	0.029	0.782
Q9	0.582	0.368
Q10	0.634	0.316
Q11	0.505	−0.124

**Table 5:** Internal validity

Item	Mean	SD	Person-centred			Care coordination		
			IIC	IDV	INFIT	IIC	IDV	INFIT
1. Discuss what’s important	2.49	0.75	0.66	0.67	0.72			
2. Involved in decisions	2.51	0.75	0.66	0.69	0.72			
3. Considered ‘whole person’	2.53	0.78	0.58	0.64	0.81			
5. Repeating information	2.47	0.76	0.34	0.51	1.26			
6. Care joined up	2.16	0.94	0.66	0.46	0.76	0.43	−0.46	0.90
7. Single named contact	1.38	1.50				0.24	0.67	1.02
8. Care planning (overall)	0.57	1.12				0.61	0.75	0.75
9. Support to self-manage	2.47	0.94	0.58	0.21	0.88	0.53	−0.21	0.68
10. Information to self-manage	2.42	0.95	0.61	0.32	0.85	0.54	−0.32	0.74
11. Confidence to self-manage	2.28	0.78	0.37	0.63	1.26			

Internal consistency was assessed through Cronbach’s Alpha and person-separation reliability. For the Person-centred scale Cronbach’s Alpha = 0.829, Person separation = 0.756. For the Coordination scale Cronbach’s alpha = 0.783, person separation = 0.672. It is possible that chronbach’s Alpha may under-estimate the internal consistency of the P3CEQ [30].

Floor effects ranged from 1.6% to 8.4% and ceiling effects ranged from 44.9% to 69.6% for all Likert-type items (data not shown); reflecting high experiences of care in our sample for some questions. The rate of missing items varied across items, although only Item 4 had a missing response rate above 15%.

### Face and ecological validity

Face validity was assessed during the conceptual development of the P3CEQ to ensure suitability of the P3CEQ for routine measurement and feedback during the development of services. A review of literature was conducted to identify existing PREMs suitable for probing P3C. Suitable measures were critically examined against core domains of P3C, and presented to stakeholders in co-design workshops to explore acceptability, utility and strengths/weaknesses [19].

Further evidence for the validity of the P3CEQ was established by use of the P3CEQ in an evaluation of a new model of care aimed at improving care coordination in adults with at least one LTC. The service redesign consisted of a joint venture of providers (hospital, general practice, social care) who share resource and risk with an integrated P3C care team [31]. The intervention focused on people with long-term conditions and included the implementation of number of elements to improve care coordination (e.g. comprehensive assessments; single care plans and points of access, multi-disciplinary team input; admission and discharge planning). The P3CEQ was able to detect significant changes between experience of care coordination at the start of the intervention (M = 8.47, SD = 3.92) and at a 1-year follow-up (M = 10.12, SD = 5.26) *t*(77) *=* 2.045, *P* = 0.044 (see Table 6). Changes in observed P3CEQ score were further confirmed through inductive qualitative analysis of semi-structured interviews with staff and patients (*N* = 21 + 26) and ethnographic observations (*N* = 12) [31]. There was a clear alignment between quantitative and qualitative data collected at each site, particularly in regards to care coordination, confirming the measures ability to measure P3C and detect changes over time in real-word healthcare settings. Coordination scores were calculated using the scoring mechanism outlined below.

**Table 6:** Means and standard deviations for each item within the coordination scale of the P3CEQ

Item	Baseline		Follow-up	
	*M*	SD	*M*	SD
joined up care	1.49	1.08	1.54	1.02
single named contact	0.81	1.34	1.15	1.47
Care plan exists	0.88	1.38	1.12	1.46
Care plan available	0.04	0.34	0.38	1.01
Care plan useful	0.68	1.06	0.81	1.08
Care plan followed	0.65	1.10	1.00	1.29
Support to self-manage	1.99	1.13	2.18	1.08
Information to self-manage	1.94	1.14	1.95	1.12
**Total**	**8.47**	**3.92**	**10.12**	**5.26**

### Correlates of P3C

Correlates of person-centred coordinated care included comparisons of responses across gender, educational level, age and housing status. The purpose of analysing correlates of person-centred care is to identify whether the findings from the measure correlate in a logical way to current understanding of the role of social context in healthcare. No *a-priori* assumptions were made, however. The results indicate that males (18.69 ± 4.74) report significantly higher levels of person-centredness than females (17.80 ± 5.20), *T*(2301) = 4.233, *P* ≤ 0.001, (see Table 7). This pattern is replicated in care coordination with males (8.54 ± 3.80) reporting more care coordination than females (7.74 ± 3.90), *T*(2301) = 4.978, *P* ≤ 0.001. There was no significant difference between age groups in the level of experienced person-centred care *F*(7 2334) = 1.174, *P* = 0.314 or care coordination *F*(7 2334) = 1.598, *P* = 0.131. Education had a significant effect on experienced person centredness *F*(5 1990) = 2.519, *P* = 0.028, with adults with no formal education and primary school education reporting lower person centredness than all other groups. Participants with primary school education reported significantly lower levels reporting significantly lower levels of person centredness than those with secondary or higher levels of education (see Table 7). A similar effect was found in care coordination, with scores being significantly lower for those with no or primary school educational attainment than those with higher levels of educational attainment *F*(5 1990) = 2.510, *P* = 0.028. There was a significant in the level of person-centred care and care coordination experienced based on housing status. Person-centred care was significantly lower in those living alone or in an institution, than those living with spouses/partners or families *F*(4 2296) = 5.886, *P* ≤ 0.001. Care coordination was lower in patients living alone or with roommates than when living with spouses/partners or family *F*(4 2296) = 6.174, *P* ≤ 0.001.

**Table 7:** Analysis of based on gender, age, education and housing status on P3C scoring.

	Person-centred	Care coordination
**Gender**		
Male	18.69 ± 4.74	8.54 ± 3.80
Female	17.80 ± 5.20	7.74 ± 3.90
*T-test*	*P < 0.001*	*P < 0.001*
*Effect size*	*4.233*	*4.978*
**Age**		
18-21	16.78 ± 5.63	6.22 + 3.03
20-39	18.59 ± 4.48	8.19 ± 3.17
30-39	18.10 ± 3.16	7.93 ± 3.38
40-49	18.02 ± 4.48	9.94 ± 3.68
50-59	17.92 ± 4.90	7.92 ± 3.66
60-69	18.46 ± 5.05	8.18 ± 3.92
70-79	18.31 ± 5.02	8.35 ± 4.01
80+	17.65 ± 5.09	7.75 ± 3.878
*ANOVA*	*P = 0.314*	*P = 0.031*
*Effect size*	*1.174*	*1.598*
**Education**		
None	15.82 ± 6.35	6.93 ± 4.29
Primary	17.13 ± 5.65	7.29 ± 4.28
Secondary	18.45 ± 4.90	8.39 ± 3.75
College	18.45 ± 4.72	7.90 ± 3.67
Undergraduate	18.13 ± 4.82	7.89 ± 3.66
Postgraduate	18.60 ± 4.97	8.60 + 3.90
*ANOVA*	*P = 0.028*	*P = 0.028*
*Effect size*	*2.519*	*2.510*
**Housing**		
Alone	17.57 ± 5.21	7.50 ± 3.88
With Spouse/partner	18.51 ± 4.83	8.36 ± 3.84
With family (not spouse)	18.8.62 ± 4.85	8.25 ± 3.64
With roomates	16.69 ± 6.29	6.23 ± 3.94
Institution	15.23 ± 6.73	8.05 ± 4.01
*ANOVA*	*P* ≤ *0.001*	*P *≤* 0.001*
*Effect size*	*5.886*	*6.174*

### Applicability

Of the 2363 participants, 7.7% had rates of missing data above 20%. All questions were answered by 67.9% of participants; 16.7% of participants had one missing data point.

### Scoring

The P3CEQ contains a combination of Likert-type scales (0–3) and dichotomous items (0, 3). Participants’ scores for the Person-centred care scale are calculated by summing all scores in the person-centred care scale (see Table 5). Care coordination scores are calculated identically to person-centred care scores, with the exception of Q8. It is recommended that Q8 should be a calculated average of scores from Q8a–Q8d to ensure the care coordination sub-scale is not weighted towards care planning. A total P3C score can be found by summing all items, Q8 should still be scored as an average of Q8a–Q8d.

## DISCUSSION

The P3CEQ was designed to measure changes in experience in response to new models of care prioritizing person-centred and coordinated care delivery [6, 32]. It was co-designed with a range of stakeholders including patients, commissioners and service delivery professionals to create a brief measure to evaluate person-centred coordinated care from the perspective of the patient [19]. It is designed to have a broad coverage of P3C while remaining concise and efficient. It probes the core domains of person-centred coordinated care (see Table 1). If the tool is used on the same cohort over time, continuity can be assessed through the combined construct of the tool.

The psychometric properties of the P3CEQ were analysed to assess the dimensionality, reliability and validity of the P3CEQ in 2363 patients with LTCs using a combination of classical and IRT based psychometrics. The partial credit Rasch Model was utilized to analyse dimensionality, while person-separation analysis was used to assess reliability. Face validity had been previously identified [19]. The measure appears to have high ecological validity with demonstrable sensitivity to change in clinical interventions. Analysis of the construct appears to logically follow current understanding of the role of the social context in care. These findings appear to indicate a reliable measure containing two unidimensional scales, with demonstrable face and ecological validity, both as a measure of person-centred care, and as a tool in the evaluation of service intervention.

Classical (true-score) analysis was also conducted alongside IRT methodologies for reference purposes, due to increased recognizability of classical methodology. There was a high level of corroboration between IRT and classical methodologies findings, for example Chronbach’s alpha, and person-separation reliabity, however IRT models are more mathematically sound and represent a more accurate assessment of the psychometric properties of the measure.

The outcome of the analysis revealed that the measure covers two closely related constructs—person-centredness and care coordination, with the PCA thereby confirming design of the instrument [19]. Internal and external validity were assessed to be sufficient through cross-dimension correlations, age, gender, education and housing status. Internal consistency was acceptable. Construct and face validity were assessed previously [19].

There was some overlap on three items in the scale (see Table 5). With these items, it is difficult to distinguish between person-centredness and care coordination due to the overlapping constructs. It was therefore decided to keep the items and include them in both scales. Rasch analysis was conducted to identify the unidimensionality of each scale and supported the inclusion of items in both person-centred care and care coordination scales. Each scale was found to be unidimensional, despite the overlap.

The decision was made to move Item 4 to the end of the measure as an optional question. The importance of the involvement of family and carers for person-centred care led to the retention of this item as an optional question (which does not contribute to the final scoring of the measure). We suspect that this question was difficult for people to answer for two separate reasons. Firstly, it combines the direct quantification of family/carer involvement in care decisions whether or not this was desired by the respondent. Secondly, for people with LTCs and MLTCs (particularly older individuals) the involvement of family/carers may not be optional. However, our patient stakeholder co-design team felt very strongly that individuals’ be given the choice of family involvement in decisions about their care, hence requirement for this question.

External and internal validity were assessed for both scales and found to be acceptable. As some items were included in both the person-centred care and care coordination scales they naturally had low IDV values, however, as previously stated, for these items it is difficult to disentangle the difference between person centred care and care coordination. The items were found to be unidimensional both as part of the person centred care and care coordination scales (see Table 5) and should therefore be considered appropriate for use within each scale.

P3CEQ data collected as part of an intervention was analysed to identify if the measure was sensitive to change in coordination. Longitudinal changes in P3CEQ scores were validated by semi-structured interviews (*N* = 47) and ethnographic observation (*N* = 12), confirming that the P3CEQ care coordination scale does appear to be sensitive to change over time in real-world settings.

The P3CEQ has also been used as a tool to drive change at a practice level by providing feedback to professionals working in new models of care across the South West. This has involved providing summary information (consisting of mean scores per question and per domain) over time to track improvement from a baseline. This has been supplied as user-friendly bar charts, used by professionals for quality improvement efforts and identification of target areas for improvement. The P3CEQ is being deployed in a number of real-world healthcare settings, where it is being used alongside our organizational change tool (P3C-OCT) [33], which provides a development process for organizations to improve/support P3C. Future work will investigate potential correlations between results of P3C-OCT (e.g. what practices identify as being done to improve P3C) and P3CEQ (e.g. patient experiences). This study used the P3CEQ to aggregate across a sub-population with LTCs. It is also potentially possible to incorporate the questionnaire as a part of the care planning process with results considered by patient and practitioner in order to drive improvement by bringing about immediate changes to individual care.

## CONCLUSION

The P3CEQ offers a patient focused perspective on the extent to which health and social care services are providing person centred coordinated care for individuals with complex care needs. It is unique in bringing together the concepts of coordination and person centredness, two domains of particular importance to individuals with complex needs. Feedback from stakeholders who are using the measure has been extremely positive, it appeals to a range of both patients and professionals due to its content, tone and brevity. This has been evidenced by the adoption of the P3CEQ in two major Horizon 2020 funded studies (SELFIE & SUSTAIN) that aim to improve integrated and tailored care for older adults across Europe, resulting in the translation of the measure into German, Dutch, Estonian, Spanish, Catalan and Norwegian. The measure is also being used in various new models of care in the UK. This paper establishes that the psychometric properties of the P3CEQ indicate a reliable and valid measure of P3C.
